# DNASE1L3 as an indicator of favorable survival in hepatocellular carcinoma patients following resection

**DOI:** 10.18632/aging.102675

**Published:** 2020-01-24

**Authors:** Shuncong Wang, Haiqing Ma, Xuemin Li, Xiangqiong Mo, Haiyu Zhang, Lewei Yang, Yun Deng, Yan Yan, Guangwei Yang, Xingwei Liu, Huanhuan Sun

**Affiliations:** 1 Department of Oncology, The Fifth Affiliated Hospital of Sun Yat-sen University, Zhuhai 519000, Guangdong, China; 2 KU Leuven, Campus Gasthuisberg, Faculty of Medicine, Leuven 3000, Belgium; 3 Guangdong Provincial Key Laboratory of Biomedical Imaging, The Fifth Affiliated Hospital, Sun Yat-sen University, Zhuhai 519000, Guangdong, China; 4 Department of General Surgery, The Fifth Affiliated Hospital of Sun Yat-sen University, Zhuhai 519000, Guangdong, China; 5 Central Laboratory, The Fifth Affiliated Hospital of Sun Yat-sen University, Zhuhai 519000, Guangdong, China

**Keywords:** DNASE1L3, hepatocellular carcinoma, TCGA, bioinformatics, prognosis

## Abstract

Hepatocellular carcinoma (HCC) is a common malignancy with a dismal prognosis. It is of great importance to identify biomarkers for the prediction of patients’ survival.

The mRNA expression level of deoxyribonuclease 1 like 3 (DNASE1L3) and its correlation with survival were accessed in 424 samples from The Cancer Genome Atlas database. Its expression level was confirmed by real-time quantitative polymerase chain reaction and western blotting in 20 pairs of postsurgical specimens. In addition, immunohistochemistry staining of DNASE1L3 was also performed in 113 postoperative samples, using a histochemistry score system. The relationship between patients’ survival and DNASE1L3 expression level was evaluated by the Kaplan-Meier method.

DNASE1L3 is downregulated in both mRNA and protein levels in HCC tissues, compared with adjacent normal tissues. 52 of 113 HCC specimens showed positive DNASE1L3 protein expression. Patients with positive DNASE1L3 expression had significantly longer overall survival, compared with patients with negative expression (*p* = 0.023). However, the DNASE1L3 fails to discriminate progression-free survival (*p* = 0.134). Multivariate COX analysis revealed that positive DNASE1L3 expression and higher differentiation were significantly associated with better overall survival.

This study demonstrated that positive DNASE1L3 expression is an independent prognostic factor for better survival in HCC patients following radical resection.

## INTRODUCTION

Hepatocellular carcinoma (HCC) represents a malignancy originating from hepatocytes mostly arising from cirrhosis, accounting for more than 90% of primary liver cancer. The global age-standardized incidence rate for HCC is 10.1 patients per 100,000 person-years, with 80% of cases occurring in East Asia and Africa [1]. An estimated 42,030 new HCC cases and 31,780 HCC-death will be observed in 2019 [2]. The World Health Organization estimated that the number of patients died from liver cancer may exceed 1 million in 2030 [3]. The risk factors for HCC include hepatitis B virus (HBV) infection, hepatitis C virus (HCV) infection, excessive alcohol intake, aflatoxin and positive family history [4]. All these factors cause deoxyribonucleic acid (DNA) damage and gene mutations of whom accumulation promotes chronic inflammation, fibrosis and eventually development of HCC [5–8]. Currently, universal adoption of HBV vaccination, implantation of direct-acting anti-HCV agents and increased incidence of metabolic syndrome and obesity are changing the etiological spectrum of HCC, especially in western countries.

Currently available curative methods for HCC include radical resection, ablation, and liver transplantation, however, only 10–30% of patients are eligible for these treatments [9]. Recent therapeutic development in HCC includes the establishment of ablation for small HCC, and sorafenib, lenvatinib, and immune checkpoint inhibitors for advanced cases [4]. However, response rate of HCC towards chemotherapy, radiotherapy, targeted therapy, and immunotherapy is relatively low, limiting their therapeutic effect in advanced cases. Despite the survival improvement benefited from above-mentioned development of treatment, liver cancer ranges the second most fatal malignancy, with a five-year survival rate of 18%, following pancreatic cancer [10, 11]. Therefore, the development of diagnosis and treatment of HCC is urgently needed, and exploring the molecular mechanism and identifying the molecules related to prognosis have far-reaching significance for improving the long-term prognosis of HCC patients.

Deoxyribonuclease 1 like 3 (DNASE1L3) is a secreted DNASE1-like nuclease that can digest DNA in chromatin, and its deficiency may lead to anti-DNA responses and autoimmunity in both humans and mice [12–15]. In addition, serum DNASE1L3 participates in the circulating plasma DNA homeostasis by enhancing fragmentation and influencing end-motif frequencies [16]. Its deficiency involves in the pathogenesis of pediatric onset systemic lupus erythematosus (SLE) by regulation of inflammasome activation and subsequent cytokine secretion and pediatric onset SLE is characterizing by positive anti-dsDNA antibody, low complement, antineutrophil antibody and a propensity for developing lupus nephritis [12, 15, 17]. Partial deficiency of DNASE1L3 in humans may also initiate hypocomplementemic urticarial vasculitis [18, 19]. Restoration of DNASE1L3 may represent a therapeutic option for SLE [12]. In recent years, studies have reported that overexpression of the DNASE1L3 gene in ovarian cancer cells can degrade the tumor cell genome and cause cell death [20]. And, the expression of DNASE1L3 is closely related to the staging of clear cell renal cell carcinoma [21]. However, the expression level of DNASES1L3 and its relationship with prognosis in HCC remain unknown. Here, we preliminarily explored the mRNA expression level of DNASE1L3 and its relationship with survival, based on the bioinformatics analyses. Additionally, we confirmed these results from bioinformatics analyses in postoperative samples in our institution by real-time quantitative polymerase chain reaction (RT-qPCR), western blotting and immunohistochemistry (IHC).

## RESULTS

### Analyses of The Cancer Genome Atlas (TCGA) data

**Figure 1:**
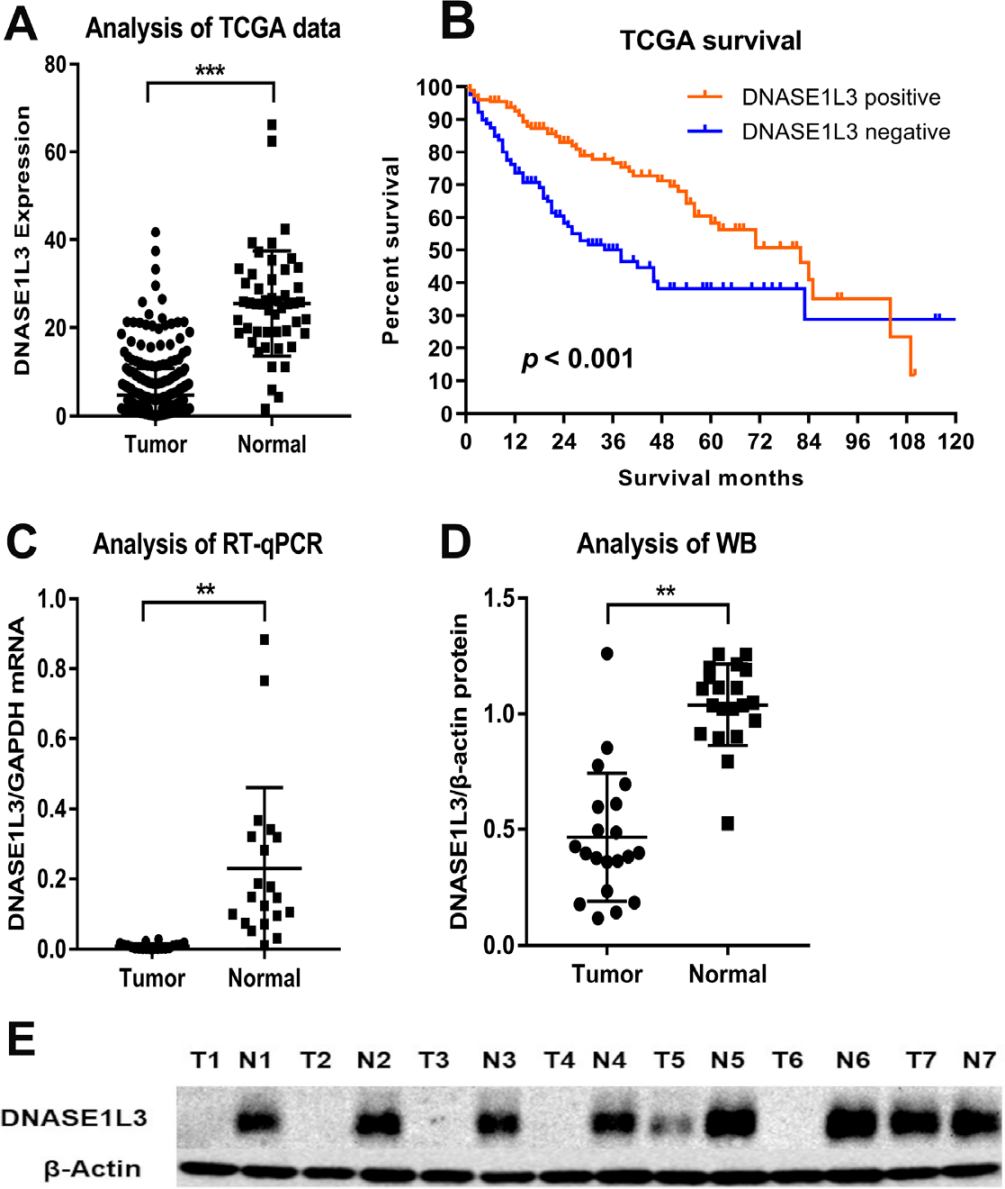
**Comparison of DNASE1L3 expression level between HCC and adjacent normal tissues.** Comparison of mRNA levels of DNASE1L3 between HCC tissues and paired normal tissues (**A**) and survival difference by mRNA levels (**B**) in the TCGA database and validation of DNASE1L3 expression level in mRNA (**C**) and protein level (**D** and **E**).

We accessed the mRNA expression level of DNASE1L3 and survival time in HCC tissues (50) and normal tissue (374) from the TCGA database. As shown, the expression of DNASE1L3 in liver cancer tissues was significantly lower than that of adjacent normal tissues (*p* < 0.001, Figure 1A). More importantly, Kaplan-Meier analysis. demonstrated that positive DNASE1L3 expression is significantly associated with longer survival time (*p* < 0.001, Figure 1B).

### Validation of the expression pattern of DNASE1L3 in HCC after resection

To further confirm the aforementioned differential mRNA expression of DNASE1L3 and its implication on prognosis in patients after radical resection for HCC (Table 1), we performed RT-qPCR, western blotting and IHC staining on postsurgical specimens. RT-qPCR further confirmed the significantly lower expression of DNASE1L3 mRNA in HCC, compared with normal tissues (*p* < 0.001, Figure 1C). Western blotting revealed that the expression level of DNASE1L3 protein was significantly lower in cancerous tissues than in adjacent normal tissues (*p* < 0.001, Figure 1D, 1E).

**Table 1:** Summary of baseline characteristics of HCC patients in current study.

**Categories**	**Cases (113)**	**Percentage (%)**
**Age (year)**		
Average	49.89	
Median	47	
Range	25 - 90	
**Sex**		
Female	13	11.50%
Male	100	88.50%
**Differentiation**		
Low	13	11.50%
Medium	74	65.49%
High	6	5.31%
Other	20	17.70%
**Tumor size (>3cm)**		
Yes	81	71.68%
No	32	28.32%
**HbSAg**		
Positive	77	68.14%
Negative	15	13.27%
Unknown	21	18.58%
**AFP**		
Elevated	60	53.10%
Normal	22	19.47%
Unknown	31	27.43%
**DNASE1L3 expression**		
Negative	61	53.98%
Positive	52	46.02%
**Survival**		
Alive	64	56.64%
Dead	42	37.17%
Loss of follow-up	7	6.19%

**Figure 2:**
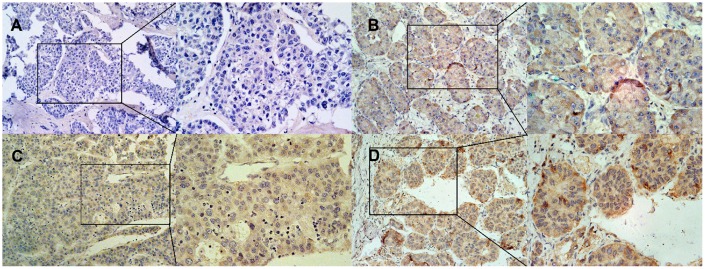
**Representative of IHC staining of DNASE1L3 in HCC.** (**A**) negative expression, (**B**) weakly positive expression, (**C**) moderately positive expression, (**D**) strongly positive expression. The left side of the **A**, **B**, **C**, and **D** pictures is 200X, and the right side is 400X.

After IHC staining, both 200-fold microscopic images and the 400-fold microscopic images were collected for analysis. DNASE1L3 is mainly expressed in the cytoplasm and cell membrane, and negative, weakly positive, moderately positive and strongly positive expression of DNASE1L3 are shown in A, B, C and D in Figure 2 respectively. 52 cases (46.02%) were showing positive expression with 61 negative counterparts (53.98%) (Table 2). Patients’ clinicopathological characteristics were summarized by expression status, with no significant difference in gender, pathological differentiation, tumor size and hepatitis B virus surface antigen (HBsAg) level between two groups (*p* > 0.05). But a significant correlation between serum alpha-fetoprotein (AFP) level before surgery and survival status and the DNASE1L3 expression level was observed (*p* < 0.05) (Table 2).

**Table 2:** Relationship between DNASE1L3 expression and clinical characteristics.

**Categories**	**DNASE1L3 Positive (n=52)**	**DNASE1L3 Negative (n=61)**	***P* value**
**Age (year)**			
Average	50.79	52.36	
Median	49	49	
Range	28-80	25-90	
**Sex**			
Female	47	53	0.561
Male	5	8	
**Differentiation**			
Low	3	10	0.269
Medium	38	36	
High	3	3	
Other	8	12	
**Tumor size (>3cm)**			
Yes	34	47	0.170
No	18	14	
**HBsAg**			
Positive	37	40	0.571
Negative	5	10	
Unknown	10	11	
**AFP**			
Elevated	29	31	0.003
Normal	9	1	
Unknown	14	30	
**Survival**			
Alive	36	28	0.043
Dead	14	28	
Loss of follow-up	2	5	

### Survival analyses by DNASE1L3 expression status and clinicopathological factors

**Figure 3:**
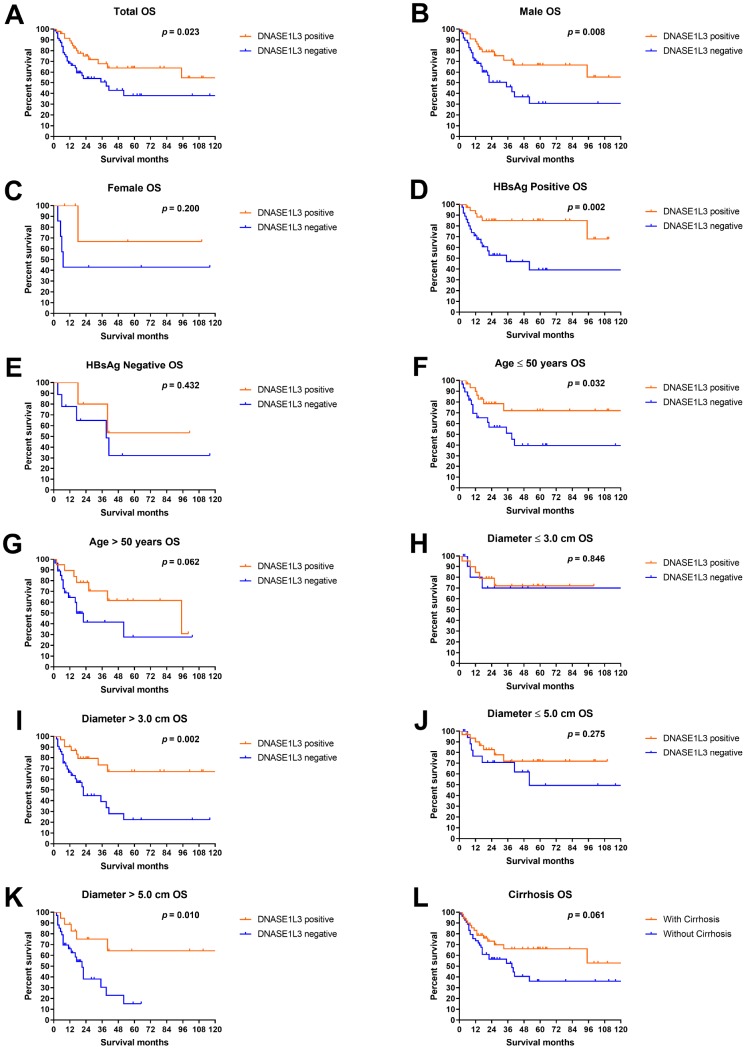
**Overall survival analyses by DNASE1L3 expression level.** Overall survival analyses according to DNASE1L3 status in all patients (**A**), male (**B**), female (**C**), patients with positive HBsAg (**D**), patients with negative HBsAg (**E**), patients not older than 50 years (**F**), patients older than 50 years (**G**), patients with HCC smaller than 3 cm in diameter (**H**), patients with HCC bigger than 3 cm in diameter (**I**), patients with HCC smaller than 5 cm in diameter (**J**), patients with HCC bigger than 5 cm in diameter (**K**) and patients with cirrhosis (**L**).

The median overall survival (OS) time of DNASE1L3 positive patients was more than 120 months, compared with 39 months in DNASE1L3 negative counterparts. HCC patients with positive expression of DNASE1L3 had a significantly better prognosis than patients with negative expression of DNASE1L3 (*p* = 0.023, Figure 3A). Subgroup analyses showed that significantly better survival in DNASE1L3 positive patients can be observed in males, patients with positive HBsAg, patients younger 50 years and patients harboring tumors greater than 3 cm or 5 cm (Figure 3). For other subgroups, a non-significantly but numerically superior survival was observed in DNASE1L3 positive patients (except for patients with a tumor smaller than 3 cm). Survival analyses by clinicopathological features showed that significant survival difference was observed in patients with different tumor sizes (5 cm as a threshold) and different pathological differentiation degrees (Supplementary Figure 1). In addition, patients with younger age, preoperational AFP level less than 200 ng/mL, normal post-operational AFP or tumor size less than 3 cm were showing a numerically but insignificantly better survival. Combinatory analyses of DNASE1L3 and other clinicopathological factors showed a promising discriminating power in predicting patients’ survival (Supplementary Figure 2). Survival analyses by DNASE1L3 and tumor size (3 cm as a threshold) showed that patients with tumors bigger than 3 cm and negative DNASE1L3 expression are associated with the worst prognosis, with the remaining patients showing similarly better survival (*p* = 0.004, Supplementary Figure 2A). And the survival trend remained similar when the threshold for size is 5 cm (*p* < 0.001, Supplementary Figure 2B). Interestingly, DNASE1L3 expression level can further discriminate patients’ survival in different pre-surgical and post-surgical AFP levels (*p* = 0.006 and *p* = 0.023 respectively, Supplementary Figure 2C, 2D). In the other analyses, DNASE1L3 can discriminate patients under each category by different clinical features (Supplementary Figure 2E–2H).

**Figure 4:**
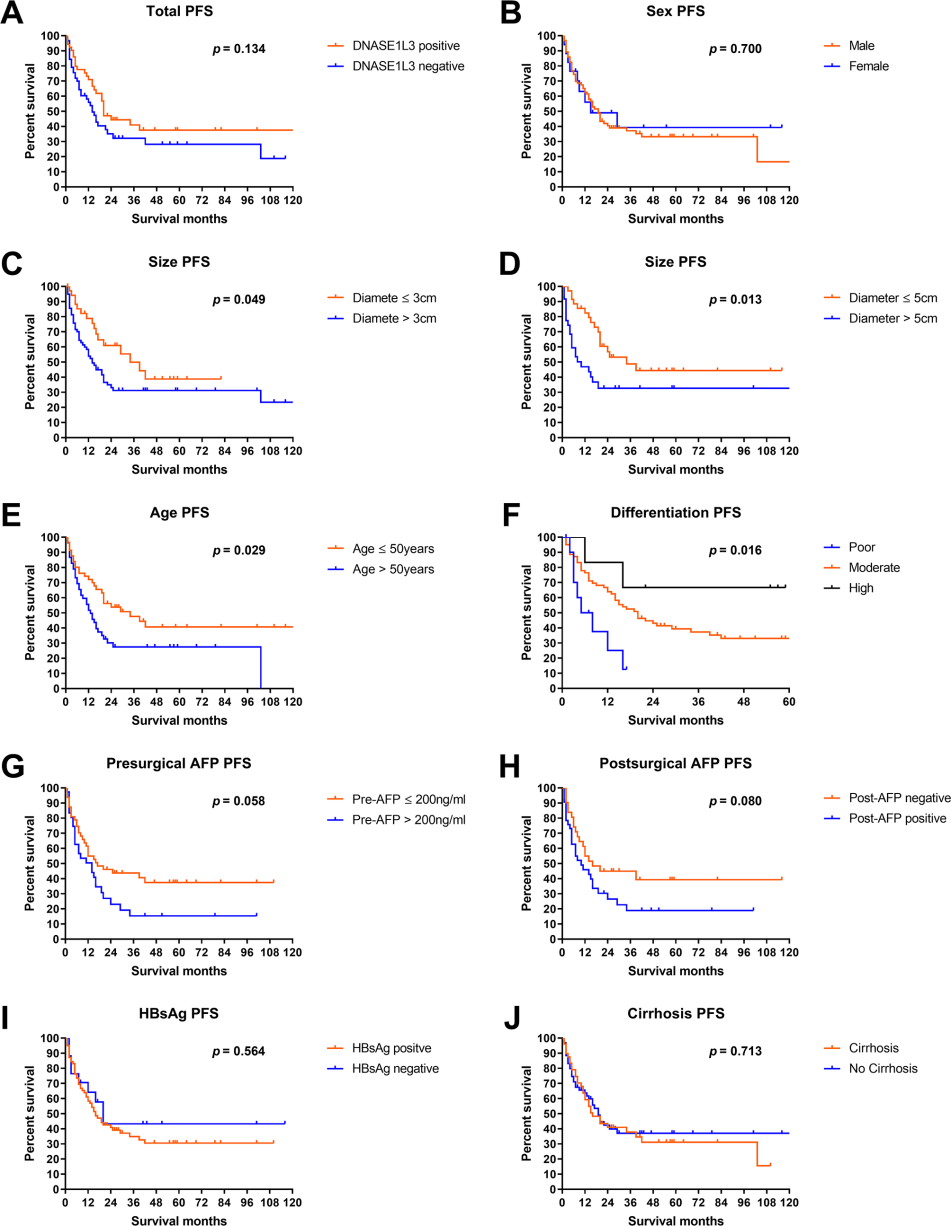
**Progression-free survival analyses by DNASE1L3 expression level.** Progression-free survival analyses according to DNASE1L3 status in all patients (**A**) or according to sex (**B**), size (**C**, **D**), age (**E**), differentiation level (**F**), presurgical AFP (**G**), postsurgical AFP (**H**), HBsAg level (**I**) and cirrhosis status (**J**).

As shown in Figure 4A, the progression-free survival (PFS) of DNASE1L3 positive patients was slightly but not significantly better than that of DNASE1L3 negative patients (*p* = 0.134). Analyses of PFS by clinicopathological characteristics showed that significant PFS difference can be seen in patients with different sizes, ages, and differentiation levels (Figure 4B–4F). And a numerically but not significantly different survival gap can be observed in the analyses by presurgical and postsurgical AFP (Figure 4G, 4H). A marginal survival gap was observed in PFS by HBsAg and presurgical cirrhosis status (Figure 4I, 4J).

### Independent risk factors for HCC

We performed Cox regression analyses to study the effect of clinical factors on patients’ OS. In the univariate Cox analysis, the differentiation level, HBsAg status, preoperative AFP level, and DNASE1L3 expression were risk factors for prognosis (Table 3). In multivariate Cox analysis, DNASE1L3 expression and differentiation level are independent risk factors for OS, with an HR of 0.463 for patients with positive DNASE1L3 expression and an HR of 0.154 and 0.047 for medium differentiation group and highly differentiated group respectively (Table 3).

**Table 3:** Univariate and multivariate Cox regression analyses for HCC patients received radical resection.

	**Univariate**			**Multivariate**		
**Categories**	**95% CI**	**HR**	***P*value**	**95% CI**	**HR**	***P*value**
**Age**						
≤50 years		1.0		-	-	
>50 years	0.842-2.834	1.545	0.16	-	-	
**Sex**						
Male		1.0		-	-	
Female	0.430-2.796	1.096	0.847	-	-	
**Differentiation**						
Low		1.0			1.0	
Medium	0.074-0.373	0.166	<0.01	0.063-0.374	0.154	<0.01
High	0.006-0.409	0.050	<0.01	0.005-0.407	0.047	<0.01
Other	0.121-0.805	0.312	0.02	0.094-0.723	0.261	<0.01
**Tumor size**						
>3cm		1.0		-	-	
≤3cm	0.239-1.235	0.543	0.15	-	-	
Unknown	0.531-4.278	1.508	0.44	-	-	
**HBsAg**						
Positive		1.0			1.0	
Negative	0.596-3.250	1.392	0.44	0.366-2.653	0.985	0.98
Unknown	1.351-5.531	2.733	<0.01	0.880-7.354	2.543	0.08
**AFP**						
Elevated		1.0				
Normal	0.401-2.305	0.962	0.93	0.563-3.708	1.445	0.44
Unknown	1.060-4.008	2.061	0.03	0.529-3.838	1.424	0.48
**DNASE1L3**						
Negative		1.0			1.0	
Positive	0.208-0.755	0.397	<0.01	0.238-0.904	0.463	0.02

## DISCUSSION

In this study, we demonstrated that the positive DNASE1L3 expression is associated with better PFS and OS in the HCC patients after radical resection. To the best of our knowledge, this is the first study to demonstrate the expression level of DNASE1L3 and its relationship with patients’ prognosis.

DNASE1L3 is a member of the deoxyribonuclease I family, which encodes proteins that cleave single-stranded and double-stranded DNA to produce a DNA fragment with a 3-OH terminus [16]. It plays a role in nuclear endosomal DNA fragmentation during apoptosis and necrosis, and mutations in this gene are thought to be related to the occurrence of SLE [12, 15, 17, 22–24]. Previous studies have reported that the serum concentration of DNASE1L3 protein is decreased in dermatomyositis, SLE, and rheumatoid arthritis, suggesting that DNASE1L3 may be involved in the development of autoimmune diseases [13]. Additionally, its role in ovarian cancer and clear cell renal cell carcinoma has been reported [20, 21]. Here, we demonstrated that DNASE1L3 is down-regulated in HCC tissues and its positive expression is associated with better survival. Differentiation level of HCC is significantly associated with the prognosis, in line with a previous study [25]. In addition, we found that the positive rate of DNASE1L3 varies among HCC with different degrees of differentiation. In poorly differentiated HCC, the positive rate was 15.38% (2/13 cases), with 51.35% (38/74) in moderately differentiated HCC and 50% (3/6) in highly differentiated HCC respectively. These preliminary data suggest that it may affect the biological behavior of cancer cells by inducing the differentiation, and ultimately alter the clinical outcome of patients. More importantly, multivariate Cox regression analyses showed that both pathological differentiation and DNASE1L3 expression are independent risk factors for prognosis, indicating that both of them can influence survival separately. Additionally, previous studies have shown that the liver is a central immunity-regulating organ that maintains immune tolerance [26]. And disorder in the liver immune network is a hallmark of chronic liver disease, and HCC shows exclusively a chronic inflammatory environment compared with other malignancies [26]. Taken together, we hypothesized that DNASE1L3 may improve the prognosis of HCC patients by regulating immune networks.

Here, we demonstrated DNASE1L3 can stratify OS in all patients and different subgroups. The superior survival discriminating power of DNASE1L3 was observed in HBsAg positive patients and this may be associated with the hydrolysis of HBV DNA by DNASE1L3 in HCC cells because, reportedly, increased HBV DNA level is associated with poorer survival in HCC patients [27]. Specifically, HBV DNA integration, a major mechanism for HCC carcinogenesis, is significantly higher in patients with positive HBsAg than those with negative HBsAg and thus the prognosis discriminating effect of DNASE1L3, which can degrade intracellular HBV DNA in HCC cells, is more evident in HBsAg positive patients [28]. The prognosis of patients with AFP ≤ 200ng/mL before surgery was better than that of patients with AFP > 200ng/mL, in accordance with a previous report [29]. AFP affects the biological behavior of HCC in a complicated manner, including inhibition of macrophages, anti-tumoral immunity of natural killer cells and T lymphocytes and regulation of cell proliferation and apoptosis [30–37]. Although survival superiority of DNASE1L3 positive patients can be seen in both males and females, significant survival difference can be only seen in males, which may be attributed to the small sample size in females (13 cases). Survival analysis by different tumor size thresholds found that significant survival superiority can only be seen in the analysis with 5 cm as a threshold rather than the analysis with 3 cm as a threshold, in line with a previous publication [38]. Our study found that there was no statistical difference in survival between sexes, in discordance with a previous report where females showed significantly better survival than male counterparts in HCC [10]. The main reason for this discrepancy is that we included operable Asian patients receiving radical surgery, compared with the previous study where the majority of HCC patients were Caucasian or African Americans, who were diagnosed with different stages (localized, regional and distant) and received different treatments, including radical surgery, liver transplantation, interventional therapy and so on. Previous research suggests that preoperative AFP level can be used to clinically assess patient outcomes and our study confirmed this trend in a more precise manner: patients with significantly elevated AFP level (AFP > 200 ng/ml) can be subdivided into DNASE1L3 positive and negative subgroups, between which there is a significant difference in prognosis [39]. In addition, DNASE1L3 can also better stratify the prognosis in patients with AFP ≤ 200 ng/ml. This indicates the synergistic discriminating power of combinatory use of DNASE1L3 and AFP and provides a basis for guiding clinical precision treatment. However, for patients with tumors with a maximum diameter of ≤ 3 cm, the expression of DNASE1L3 failed to do so, and therefore for these patients, it is of great necessity to further identify alternative prognostic biomarkers. The analysis of PFS found that patients with positive expression of DNASE1L3 had slightly better PFS than those with negative expression. This suggests that DNASE1L3 may inhibit tumor recurrence and metastasis, but the underlying molecular biological mechanism remains unknown.

Despite novel and encouraging findings, this study should be interpreted in the context of limitations. First, due to the small sample size, some subgroup analyses only showed numerically but not significantly statistical difference; Second, the included cases are mainly residents in south China and therefore it should be conservative to interpret these results in regions other than southern China. Third, the mechanism how DNASE1L3 regulates signaling pathways and ultimately affects the survival was not explored, which necessitates both *in vivo* and *in vitro* studies. In addition, it is unknown whether the low expression of DNASE1L3 is a molecular biological event that induces HCC carcinogenesis or merely a difference caused by HCC. Fourth, the HBV-DNA copy number, preoperative liver function, and tumor staging are scant in current study.

This study demonstrates for the first time the low expression of DNASE1L3 in HCC compared with adjacent normal tissue and its predictive power in patients’ OS and PFS.

## MATERIALS AND METHODS

### *In silico* analysis

The clinical information and genomic matrix file of HCC patients were downloaded from The Cancer Genome Atlas (TCGA) database. The fragments per kilobase of transcript per million mapped reads (FPKM) value of DNASE1L3 gene in 424 samples of liver hepatocellular carcinoma (LIHC) was obtained from the data port (https://tcga-data.nci.nih.gov/tcga/) (including 374 cases of HCC tissue, 50 normal cases). In addition, 370 HCC patients with follow-up data were included in survival analysis.

### Patients and samples

We collected 20 pairs of cancer tissues and corresponding normal adjacent tissues from May 2016 to September 2016 from the First Affiliated Hospital of Sun Yat-sen University, with 11 pairs from male patients and 9 pairs from female patients. The average age is 47.1, with the corresponding standard deviation of 7.81. In addition, a total of 113 postsurgical HCC samples between August 2004 and April 2015 were collected from the Fifth Affiliated Hospital of Sun Yat-sen University, as shown in Table 1. The median follow-up time was 19 months. In the above cases, HCC was diagnosed by pathology, and patients with either liver metastatic tumors or extrahepatic metastasis have been excluded.

### mRNA extraction and RT-qPCR analysis

Total RNA of the 20 pairs of tissues was extracted using a TRIzol (Invitrogen, USA). RNA was quantified by spectrophotometry on a NanoDrop 2000 (Thermo Scientific, USA). A total of 2 μg RNA was subjected to cDNA synthesis using the Transcriptor First Strand cDNA Synthesis Kit (Roche, Switzerland). RT-qPCR was performed with the SYBR Premix Ex TaqII (Invitrogen, USA). Data were collected with the Realplex Real-Time PCR System (Eppendorf, Germany) according to the manufacturer’s instructions. The RT-qPCR gene specific primers were as follows: DNASE1L3: forward primer: 5'-CTGCTGCTTCTCCTCCTCTCCAT-3', reverse primer: 5'-AGATCCTGTTGTTGCTGTCCTTGATT-3'; GAPDH: forward primer: 5'-TGCACCACCAACTGCTTAGC-3', reverse primer: 5'-GGCATGGACTGTGGTCATGAG-3'. The mRNA expression level of DNASE1L3 in cancer tissues and paracancerous tissues was compared by a relative quantitative analysis method (2^-Delta Ct^ method), wherein Delta Ct of each sample equals average Ct value of the target gene minus average Ct value of the internal reference gene.

### Protein extraction and western blotting

Total protein was extracted from the 20 pairs of HCC and paracancerous tissues with RIPA lysis buffer (Solarbio, Beijing, China) and protein concentration was determined using a Pierce™ BCA Protein Assay Kit (Thermo Fisher, USA). Then the samples were separated by Mini-PROTEAN® Tetra Cell System (Bio-Rad, USA) and were transferred to the PVDF membrane using the Bio-Rad Criterion System (Bio-Rad, USA). Membranes were blocked with 5% non-fat dry milk in PBS containing 0.1% Tween-20 (0.1 % TBST, pH = 7.4) for 1 h. Membranes were incubated with antibodies specific for either human DNASE1L3 (rabbit polyclonal antibody, 1:400 dilutions; Thermo Fisher, USA) or beta-Actin (rabbit polyclonal antibody, 1:1,000 dilution; Thermo Fisher, USA) overnight at 4 °C. After 3 times of washing with 0.1 % TBST for 5 min, horseradish peroxidase-conjugated goat anti-rabbit secondary antibodies (1:10,000 dilution; Pierce, Thermo Fisher, USA) were applied, followed by washings with 0.1 % TBST for 5 min at room temperature. The bound immunocomplexes were detected using ECL+ reagent (Millipore, USA) with a Mini Chemi 500 system (Sage Creation Science, Beijing, China).

### Immunohistochemistry staining

Tissue sections were deparaffinized in xylene and rehydrated with a subsequent ethanol series. The sections were unmasked with antigen retrieval buffer (MVS-0099, Maxim EDTA buffer, pH 8.0) in an autoclave for 10 min at 120°C. To block endogenous peroxidase activity, the sections were treated with 0.3% hydrogen peroxide for 30 min and subsequently washed with phosphate-buffered saline. After being washed with phosphate-buffered saline, the sections were incubated with the DNASE1L3 antibody at a 1:600 dilution overnight at 4°C. The sections were then washed 3 times with wash buffer for 5 min each time. The secondary antibody of the EliVision Plus kit detection system and the enhanced polymer 3, 3′diaminobenzidine detection kit (Maxim Biotech, Fuzhou, China) were used according to the manufacturer’s instructions. After staining, the sections were washed in distilled water and dehydrated in graded alcohol.

The staining was scored in five randomly selected areas containing tumor cells, which showed membranous and cytoplasmic staining. The percentage of positive tumor cells was graded on a scale of 0-4: 0 (< 1%), 1 (1-10%); 2 (11-50%); 3, (51-70%); and 4 (> 70%). The intensity of staining was scored as follows: 0 (no staining), 1 (weak staining), 2 (moderate staining), and 3 (strong staining). The H-score, ranging from 0 to 12, was calculated by multiplying the percentage of positive tumor cells by the intensity of staining on the tissue sections. The H-scores were categorized as follows: 0: negative (-), 1-4: weak positive (+), 5-8: moderately positive (++), 9-12: strong positive (+++).

### Statistical analyses

Analysis of TCGA data was based on R x64 3.4.3 and the edgeR package, and differences in gene expression were tested by Wilcox tests with a cutoff of *p* < 0.001 [40]. Other statistical analyses were performed using SPSS 21.0 (IBM, Armonk, NY, USA). The difference between the count variables was compared by a chi-squared test. Kaplan-Meier analyses were used to compare the survival differences between groups, with log-rank t-tests for assessment of difference. The relationship between clinicopathological features and prognosis was detected by Cox regression analysis. A two-sided p-value less than 0.05 was considered as statistically significant. All images in this article were drawn in GraphPad Prism 7.0 (La Jolla California, USA).

## Supplementary Material

Supplementary Figures
