# Supplementary material for: DNASE1L3 as an indicator of favorable survival in hepatocellular carcinoma patients following resection
Source: Aging (Albany NY). 2020 Jan 24;12(2):1171–85. doi: 10.18632/aging.102675 (PMC7053625; doi:10.18632/aging.102675)
Supplement: Supplementary Figures [file aging-12-102675-s001..pdf]

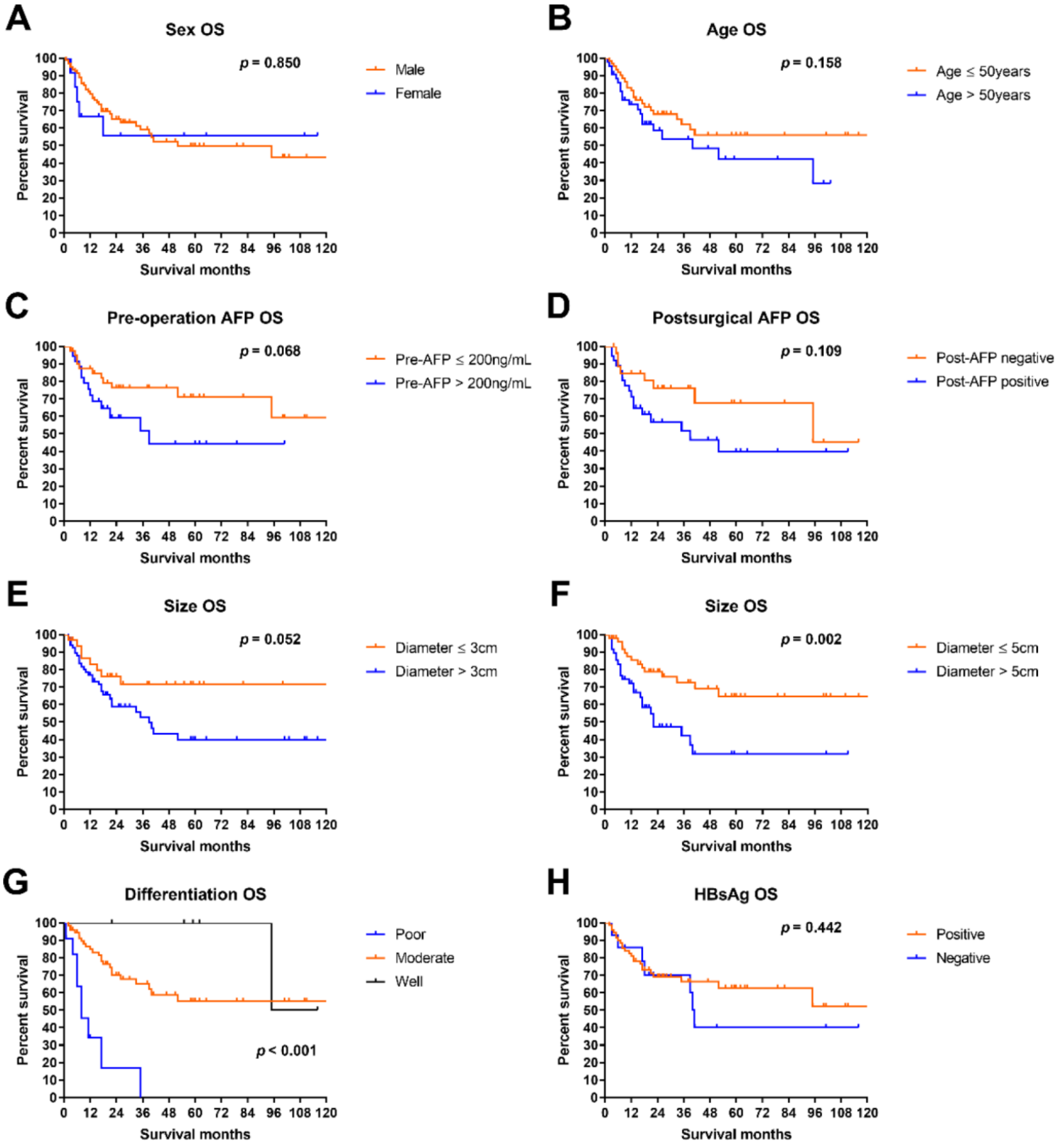

Supplementary Figure 1. Overall survival analyses of HCC patients by clinicopathological factors. Overall survival analyses of HCC patients after surgical resection by sex (**A**), age (**B**), pre-operation AFP level (**C**), postsurgical AFP level (**D**), size (**E** and **F**), differentiation level (**G**) and HBsAg (**H**).

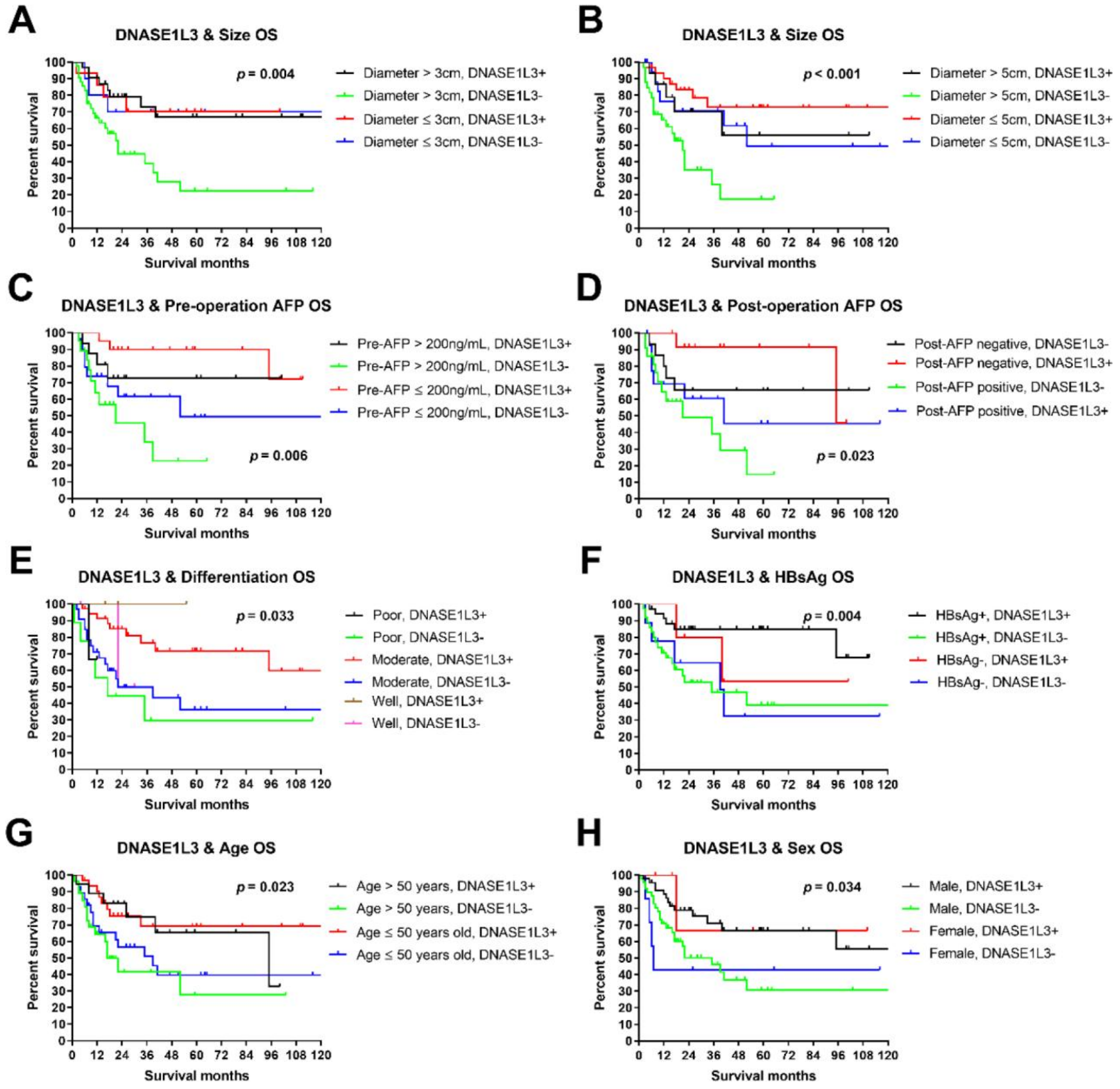

Supplementary Figure 2. Overall survival analyses of HCC patients by DNASE1L3 and clinicopathological factors. Overall survival analyses of HCC patients after surgical resection by DNASE1L3 expression and size (**A** and **B**), pre-operation AFP level (**C**), post-operation AFP level (**D**), differentiation level (**E**), HBsAg (**F**), age (**G**) or sex (**H**).
